# Prevalence and determinants of hypertension in rwanda: a secondary data analysis using the WHO STEPS survey 2022

**DOI:** 10.1038/s41598-025-14138-1

**Published:** 2025-08-23

**Authors:** Jean Damascene Hagenimana, Kagisha Divine Pascaline, Felix K. Rubuga, Kelly Mwiza, Sabine Musange F., Nathalie Umutoni, Caroline Mudereri, Jocelyne Uwitonze, Emmanuel Christian Nyabyenda, Piero I. Mazimpaka, Olivier N. Wane, Eric remera, Uwimana Aline, Marie Rosette NAHIMANA, Jeanine Condo, Gashaija Absolomon

**Affiliations:** 1 Center for Impact, Innovation and Capacity Building in Health Information Systems and Nutrition (CIIC-HIN), Kigali, Rwanda; 2 Department of community health, school of public health, university of Rwanda, Kigali, Rwanda; 3 Rwanda Biomedical Center, Kigali, Rwanda; 4 World Health Organization (WHO), Kigali, Rwanda; 5 Tulane School of Public Health and Tropical Medicine, New Orleans, LA USA

**Keywords:** Hypertension, Prevalence, Risk factors, Rwanda, WHO STEPS survey, Cardiovascular health, Diseases, Health care

## Abstract

Hypertension is a major public health concern and a leading risk factor for cardiovascular diseases in Rwanda. While previous national surveys have documented its burden, updated analysis of current prevalence patterns and determinants is needed to inform contemporary prevention strategies. This study aimed to assess hypertension prevalence and key determinants using data from the 2022 Rwanda WHO STEPS Survey to inform targeted prevention and intervention strategies. This secondary data analysis utilized nationally representative data from the 2022 Rwanda WHO STEPS Survey. Bivariate analysis was conducted using weighted chi-square tests to examine associations between hypertension and sociodemographic, behavioral, and clinical variables. Variables with *p* < 0.05 in bivariate analysis were included in a weighted multivariable logistic regression model to identify independent factors associated with hypertension. Data analysis was performed using STATA version 17, accounting for the complex survey design. Bivariate analysis was conducted using the chi-square test to assess associations between hypertension and independent variables. Significant variables were included in a multivariable logistic regression model to identify independent factors associated with hypertension. The overall prevalence of hypertension was 16.8%, with higher rates observed among older individuals and those with obesity. In multivariable logistic regression analysis, compared with younger adults, respondents aged 60–69 years were 5.7 times more likely to have hypertension (AOR = 5.7, 95% CI [3.76–8.65], *p* < 0.001). Overweight and obese individuals were 1.6 times more likely to have hypertension (AOR = 1.6, 95% CI [1.20–2.00], *p* = 0.001). Alcohol consumption in the past 12 months was associated with increased hypertension risk (AOR = 1.4, 95% CI [1.11–1.87], *p* = 0.006). Geographic disparities were noted, with individuals in the Northern (AOR = 1.5, 95% CI [1.05–2.09], *p* = 0.025), Western (AOR = 1.6, 95% CI [1.21–2.24], *p* = 0.002) and Southern (AOR = 1.7, 95% CI [1.26–2.27], *p* < 0.001) provinces at greater risk compared to the Eastern province. Hypertension is prevalent in Rwanda, with age, obesity, alcohol consumption, and regional disparities emerging as key risk factors. These findings underscore the urgent need for targeted public health interventions, including hypertension screening, weight management programs, and lifestyle modifications. Strengthening community-based prevention initiatives and policy-driven strategies can improve cardiovascular health outcomes in Rwanda.

**Supplementary Information:**

The online version contains supplementary material available at 10.1038/s41598-025-14138-1.

## Introduction

Hypertension is one of the major global risk factors for death and disability. Between 1990 and 2019, the number of people with hypertension (blood pressure of ≥ 140 mmHg systolic or ≥ 90 mmHg diastolic or on medication) doubled from 650 million to 1.3 billion^1^. Hypertension is a serious noncommunicable disease that affects both urban and rural populations equally^2^. The global noncommunicable disease (NCD) monitoring framework, endorsed by the World Health Assembly in 2013, defines elevated blood pressure as systolic blood pressure ≥ 140 mmHg and/or diastolic blood pressure ≥ 90 mmHg, regardless of whether a person is taking medication. Using this definition, the global prevalence of increased blood pressure in adults aged 18 years and above was approximately 22% in 2014^3^. If left unmanaged, hypertension can lead to serious complications, including myocardial infarction, stroke, heart failure, and renal failure. It is estimated to cause 9.4 million deaths annually worldwide and accounts for 54% of all strokes and 47% of ischemic heart disease cases^4^. The burden of hypertension is particularly significant in low- and middle-income countries, where two-thirds of the approximately 1.3 billion people with hypertension reside, hindering economic growth and productivity^2^. The prevalence of hypertension varies considerably by region and income level. In high-income countries such as Australia, Canada, and the UK, increased blood pressure is relatively uncommon (less than 13% among women and under 19% among men). In contrast, in certain Central and Eastern European countries like Croatia and Hungary, the prevalence exceeds 35% among men^5^. In northern and western African countries, particularly Morocco, rates of uncontrolled hypertension are alarmingly high, reaching 73% among hypertensive patients in 2017. Additionally, in some West African countries, the prevalence of increased blood pressure can exceed 33% among women^6^.

Hypertension is widespread in sub-Saharan Africa, which has the highest prevalence rate globally, affecting 46% of adults aged 25 and older^7^. A cross-sectional study across four sub-Saharan African countries reported an overall age-standardized prevalence of 25.9%^8^. The region’s population is experiencing increased longevity and westernization, transforming hypertension from a rare condition to a significant public health challenge. This epidemiological transition is concerning because healthcare systems remain inadequately equipped to manage the growing burden, with limited access to treatment and poor control rates^3^. In Rwanda specifically, hypertension has emerged as a significant public health concern, mirroring regional trends. The national 2012–2013 STEP survey identified 15.9% of participants as having elevated blood pressure (systolic BP ≥ 140 mmHg or diastolic BP ≥ 90 mmHg), with rates rising to nearly 40% among those aged 55–64^9^. Despite this documented burden, significant knowledge gaps remain in understanding the current epidemiological landscape of hypertension in Rwanda. First, the most recent comprehensive national data is nearly a decade old, limiting our understanding of contemporary prevalence patterns and trends. Second, while previous studies documented baseline prevalence, they provided limited insight into the relative contribution of various risk factors specific to the Rwandan context. Third, there is insufficient evidence regarding geographic and demographic variations in hypertension burden within the country, which is essential for targeted intervention planning^10,11^. These knowledge gaps hinder the development of context-specific prevention and control strategies. This study addresses these gaps by analyzing current data from the 2022 Rwanda WHO STEPS Survey to provide updated prevalence estimates and identify key determinants of hypertension across different population segments. By identifying the most significant modifiable risk factors and vulnerable populations, this research will inform evidence-based policy development and resource allocation for hypertension prevention and control in Rwanda.

The proportion of Disability-Adjusted Life Years ( DALYs) lost due to hypertension was 2.81%, whereas mortality from hypertensive heart disease accounted for 1.82% in 2017^12^. A national strategy or plan targeting NCDs, Cardiovascular Diseases (CVDs), and their associated risk factors has been developed, with a dedicated budget for implementation. The government has established a single project implementation unit within each ministry. Rwanda also has a national surveillance system that monitors CVDs and their risk factors. Collaborative NCD intervention projects, including those addressing CVDs, have been carried out through partnerships between the Ministry of Health, other non-health ministries, and civil society organizations. Since 2019, Rwanda has been one of the priority countries involved in the United Nations-initiated ‘Defeat-NCD Partnership,’ which aims to support four areas related to NCDs: national capacity building, community health scale-up, the marketplace, and financing^12^. This study aimed to assess the prevalence and key determinants of hypertension using data from the 2022 Rwanda WHO STEPS Survey. Specifically, the study sought to: (1) determine the current prevalence of hypertension among Rwandan adults, and (2) identify sociodemographic, behavioral, and physiological factors associated with hypertension to inform targeted prevention and intervention strategies.

### Methods

This study is a secondary analysis of data from the 2022 Rwanda STEPs Survey on Noncommunicable Disease Risk Factors. The methodology includes the study approach and data source, study design and settings, sampling methodology, and data collection procedures, was commissioned by the Rwanda Ministry of Health and the Rwanda Biomedical Center, and coordinated by the University of Rwanda, College of Medicine and Health Sciences^8,13^. All methodological details presented herein are based on the original implementation of the survey, and the dataset was used with appropriate permission and acknowledgment.

### Study approach and data source

The 2022 Rwanda NCD Risk Factor Survey followed the WHO STEPwise approach, a standardized framework for NCD surveillance implemented in three sequential steps.

#### Step 1

Participants completed a structured questionnaire administered by trained personnel, collecting information on behavioral risk factors including tobacco and alcohol use, dietary habits (e.g., fruit, vegetable, and oil consumption), physical activity, and self-reported history of blood pressure, diabetes, and injuries. Data were recorded electronically using personal digital assistants.

#### Step 2

Physical measurements were taken, including blood pressure, height, weight, waist and hip circumference, and heart rate.

#### Step 3

Biochemical assessments were conducted to measure fasting blood glucose, total cholesterol, and urine albumin levels.

### Study design and settings

The study employed a cross-sectional, household-based survey design, following the World Health Organization’s (WHO) STEPwise approach to surveillance of NCD risk factors (STEPS). Data collection occurred between November 2021 and January 2022. Data were collected from all 30 administrative districts of Rwanda, ensuring urban and rural representation. This study used data from the 2022 Rwanda NCD Risk Factors Survey, which employed a nationally representative sample.

### Sampling strategy and sample size

The sampling frame used for the study was compiled by NISR, drawing from the 2012 national census and 2019–2020 DHS data. It included detailed information on village-level population distributions and was stratified by province and urban/rural classification. A multi-stage cluster sampling method was utilized to ensure representativeness across all provinces and urban–rural settings:

#### Stage 1

A total of 400 enumeration areas (EAs) were selected using probability proportional to size (PPS), comprising 280 rural and 120 urban EAs.

#### Stage 2

In each selected EA, 15 households were chosen using simple systematic sampling.

#### Stage 3

From each selected household, one eligible respondent aged 18–69 years was randomly selected using the STEPS app algorithm. This methodology ensured representativeness by age, sex, location (urban/rural), and province.

The sample size for the NCD risk factor study was determined using a standard cluster sampling formula and was calculated as follows:

n=$$\:\frac{{Z}^{2\:}p\left(1-p\right)}{{e}^{2}}$$.

where:

Z = 1.96 (95% confidence level),

*P* = 0.50 (estimated prevalence),

e = 0.05 (margin of error).

Assuming prevalence (P) = 0.50, Z = 1.96 (for 95% CI), and margin of error (e) = 0.05.

The initial estimated sample size for the study was *n* = 384.16, based on standard cluster sampling methodology. This calculation assumed a 95% confidence level, 5% margin of error, and a conservative estimated prevalence of 50% for key noncommunicable disease (NCD) risk factors. To enhance the study’s representativeness, the sample size was adjusted to account for the design effect (1.5) due to the multistage cluster sampling approach and age–sex stratification using eight distinct age brackets ranging from 18 to 69 years. This adjustment resulted in a revised sample size of 4,610 participants. Considering an anticipated non-response rate of 20%, the sample size was further increased to 5,762 to ensure sufficient data retention. Additionally, oversampling in urban areas was introduced to ensure robust urban representation, adding approximately 230 more respondents. This brought the final target sample size to 6,000 participants.

The sample was distributed across 400 Enumeration Areas (EAs) nationwide, with 280 clusters in rural areas and 120 in urban areas, reflecting Rwanda’s predominantly rural population. However, given the higher expected non-response rate in urban areas and to strengthen analytical power, the urban sampling “power” was increased to 0.46. As a result, 30.5% of all sampled clusters were ultimately allocated to urban settings, enhancing the reliability of urban-rural comparisons in the analysis.

### Inclusion criteria

Eligible participants included males and females aged 18–69 years, residing in selected households for at least three months prior to the study, and speaking either Kinyarwanda or English. Respondents were selected randomly from within each household using the STEPS app.

### Exclusion criteria

Individuals with mental or cognitive impairments affecting their ability to respond, those with severe physical disabilities (e.g., hearing or speech impairments) who made verbal participation unfeasible were excluded from the study. Although persons with disabilities are recognized as an important sub-population for NCD research, they were not targeted specifically in this survey due to design limitations and lack of trained personnel (e.g., sign language specialists). A separate, focused study is recommended for this group.

### Data collection procedures

An advance team of data collectors first visited each enumeration area (EA) to list eligible household members and gather basic demographic information, which was later used to cross-verify field data and minimize bias. In each selected household, one eligible respondent aged 18–69 years was randomly chosen using the STEPS app on password-protected Android tablets. Interviewers introduced the study, confirmed eligibility, and obtained both oral and written informed consent using forms available in English and Kinyarwanda. For participants selected for Step 3 (biochemical measurements), fasting instructions were provided during the consent process. Data collection took place from November 2021 to January 2022 across all 30 districts of Rwanda, conducted by 16 teams, each comprising three data collectors, one laboratory technician, and one supervisor. Steps 1 and 2 (structured interviews and physical measurements) were conducted at the participant’s home, while Step 3 was carried out at designated gathering points identified by local community leaders. Interviews were conducted privately. If a selected respondent was unavailable after three visits or declined participation, the household was skipped. Data were collected electronically in anonymized form, while household listings were securely stored at the Rwanda Biomedical Center (RBC). Field supervisors and data managers performed daily quality checks to ensure consistency, compliance with protocol, and data integrity.

### Data collection and data management

The data for the 2022 Rwanda STEPs Survey were collected between October 2021 and January 2022, with field work conducted across all 30 districts of Rwanda. Data were collected using the eSTEPS system on Android tablets to ensure accuracy and eliminate routing and data entry errors. This system allowed real-time validation of responses, secure storage, and encrypted transmission of data. All survey tools were translated into Kinyarwanda and culturally validated during training. Tablets simplified logistics, eliminated manual data entry, and enabled efficient, centralized oversight by the STEPS Coordinating Committee. Remote data submission ensured timely monitoring and enhanced data quality control.

### Data analysis

We conducted a secondary data analysis of all participants in the STEP survey aged 18–69 years. The study collected data from 5,776 participants across all 30 districts of Rwanda (response rate: 96.3%, *n* = 6000). Biochemical measurements followed, with 5,512 participants completing Step 3 (95.4% retention from Steps 1–2), leading to a final analytical sample of 5,676 respondents. Data cleaning and analysis were performed using STATA version 17, which addressed missing values, outliers, and inconsistencies. Descriptive analysis was used to summarize the respondent’s characteristics and the prevalence of hypertension. Univariate analysis was used to examine each variable independently, reporting proportions for categorical variables and distributions for continuous variables. Bivariate analysis was conducted using weighted Pearson’s chi-square test to examine the association between each independent variable (categorical variables) and hypertension status. A significance threshold of *p* < 0.05 was used to identify variables for inclusion in the weighted multivariable model. Model validation included assessments of goodness-of-fit, multicollinearity, and regression assumptions.

### Ethical considerations

The Rwanda NCD Risk Factors Study involved human subjects, including the collection of biological samples and physical measurements. The study protocol was reviewed and approved by the Rwanda National Ethics Committee (RNEC) under Ref No. 553/RNEC/2021 on May 18, 2021. All participants received a plain language information sheet explaining the study’s purpose, procedures, risks, and benefits. Prior to participation, each individual provided both oral and written informed consent. Specifically, for participants selected to undergo biochemical measurements (Step 3), a distinct section of the consent form was signed to authorize the collection of blood and urine samples. Consent forms were translated into English, Kinyarwanda, and French, and participants were informed of their right to withdraw at any time without consequences.

## Results

### Sociodemographic characteristics of the respondents

Among the 5,676 respondents, most were aged 30–44 years (41.9%, *n* = 2,383), whereas a smaller proportion were aged 60–69 years (12.2%, *n* = 690). The majority resided in rural areas (79.9%, *n* = 4,535) and were female (62.5%, *n* = 3,546). Nearly half had completed primary education (49.2%, *n* = 2,793), whereas 36.4% (*n* = 2,063) had no formal education. Additionally, most were married (64.1%, *n* = 3,637) and employed (85.1%, *n* = 4,831). As shown in Table 1.

**Table 1:** Respondents’ sociodemographic characteristics (*n* = 5,676).

Variable	Frequency (*n*)	Percentage (%)
Age group		
18–29	1,310	23.1
30–44	2,383	41.9
45–59	1,293	22.8
60–69	690	12.2
Province		
Kigali	613	10.8
South	1,515	26.7
West	1,313	23.1
North	925	16.3
East	1,310	23.1
Location		
Urban	1,141	20.1
Rural	4,535	79.9
Sex		
Male	2,130	37.5
Female	3,546	62.5
Education level		
None	2,063	36.4
Primary School	2,793	49.2
Secondary and High School	820	14.5
Marital Status		
Never married	942	16.6
Currently married	3,637	64.1
Separated	1,097	19.3
Employment status		
Employed	4,831	85.1
Unemployed	845	14.9

### Lifestyle and behavioral factors

The majority of the respondents were nonsmokers (*n* = 5,170; 91.1%) and had consumed alcohol in their lifetime (*n* = 4,544; 80.1%). More than half had consumed alcohol in the past 12 months (*n* = 3,245; 57.2%) and more than two-thirds were engaged in binge drinking (*n* = 3,984; 70.2%). Most of respondents had a history of blood pressure measurement (*n* = 3,134; 55.2%) and active transport (*n* = 5,476; 96.5%). With respect to physical activity and diet, most of the study participants were physically active (*n* = 5,401; 95.2%), but the majority had inadequate vegetable consumption (*n* = 5,196; 91.5%). The normal BMI was preponderant, (*n* = 3,926; 69.2%), while a smaller proportion was overweight and obese (*n* = 1,171; 20.6%) or underweight (*n* = 579; 10.2%), Most (85.3%, *n* = 4,841) did not engage in vigorous physical activities, as detailed in Table 2.

**Table 2:** Respondents’ lifestyle and behavioral factors (*n* = 5,676).

Variable	Frequency (*n*)	Percentage (%)
Current tobacco use		
No	5,170	91.1
Yes	506	8.9
Ever consumed alcohol		
No	1,132	19.9
Yes	4,544	80.1
Alcohol Consumption in the Past 12 Months		
No	2,431	42.8
Yes	3245	57.2
Blood Pressure Measurement History		
No	2,542	44.8
Yes	3,134	55.2
Active Transport (Walking/Bicycle Use)		
Yes	5,476	96.5
No	200	3.5
Perceived Importance of Salt Reduction		
Very important	1,145	20.2
Somewhat important	3,589	63.2
Not at all important	942	16.6
Fruit Consumption Frequency		
None	1,937	34.1
1–3 days	3,012	53.1
4–7 days	676	11.9
Do not know	51	0.9
Physical Activity Level		
Physical Inactive	275	4.8
Physically active	5,401	95.2
Binge drinking		
No	1,692	29.8
Yes	3,984	70.2
Heavy drinking		
No	5,221	91.9
Yes	455	8.0
BMI Status		
Underweight	579	10.2
Normal Weight	3,926	69.2
Overweight/Obese	1,171	20.6
Vegetable consumption		
Inadequate	5,196	91.5
Adequate	480	8.5
Engagement in Vigorous Physical Activity		
	835	14.7
	4,841	85.3

### Hypertension, biochemical measurements and medical history

**Fig. 1:**
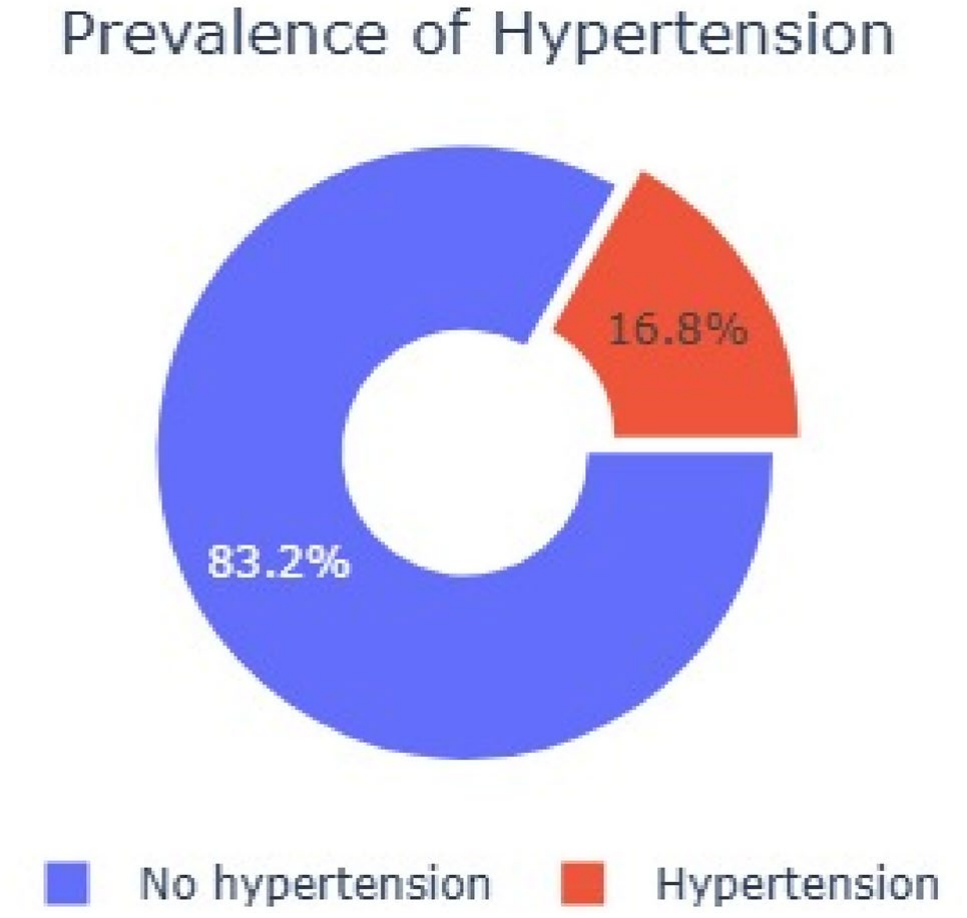
Prevalence of hypertension.

The prevalence of hypertension among respondents was 16.8% (95% CI: 15.9–17.8; *n* = 955),

Among the 5,676 respondents, diabetes was reported in (16.8%, *n* = 955), while blood glucose levels (78.1%, *n* = 4,431) and normal total cholesterol levels (97.8%, *n* = 5,549) were reported. A small proportion had a history of stroke (1.1%, *n* = 62) or heart attack/angina (4.7%, *n* = 267). Cholesterol measurement was rare (2.3%, *n* = 128), and recent cholesterol treatment was minimal (0.2%, *n* = 9). as detailed in Table 3. The prevalence of hypertension among 5,676 respondents was 16.83% (95% CI: 15.9–17.8; n=955/5,676), as detailed as shown in Figure 1.

**Table 3:** Respondents’ biochemical measurements, health outcomes, and medical history factors (*n* = 5,676).

Variable	Frequency (*n*)	Percentage (%)
Hypertension		
No	4,721	83.2
Yes	955	16.8
Diabetes status		
Nondiabetic	5,413	95.4
Diabetic	263	4.6
Blood Glucose Levels		
Low	1,111	19.6
Normal	4,431	78.1
High	134	2.4
Total Cholesterol Levels		
Normal	5,549	97.8
Borderline high	89	1.6
High	38	0.7
History of Stroke		
Yes	62	1.1
No	5,614	98.9
History of Heart Attack or Angina		
Yes	267	4.7
No	5,409	95.3
Cholesterol Measurement History		
No	5,548	97.7
Yes	128	2.3
Recent Cholesterol treatment		
No	5,667	99.8
Yes	9	0.2

### Bivariate analysis

The bivariate analysis revealed significant associations between hypertension and several factors, including age (*p* < 0.001), residence (*p* = 0.001), urban/rural residence (*p* = 0.026), education level (*p* = 0.003), marital status (*p* < 0.001), employment status (*p* = 0.011), alcohol consumption (*p* = 0.001), alcohol consumption in the past 12 months (*p* = 0.008), excessive drinking (*p* = 0.004). Factors such as sex, current tobacco use, active transport (walking/bicycle use), perceived importance of salt reduction, fruit consumption, physical activity level, history of stroke, history of heart attack, vegetable consumption, and recent cholesterol treatment were not significantly associated with hypertension (*p* > 0.05). The detailed results are shown in supplementary table 1

**Table 4:** Determinants of hypertension among respondents (n=5,676).

Variable	COR [95%CI]	AOR [95%CI]	P-value
Age category			
18-29	*Ref*	*Ref*	
30-44	2.0[1.528-2.715]	1.9[1.354-2.633]	<0.001
45-59	4.7[3.468-6.318]	4.1[2.848-6.015]	<0.001
60-69	7.3[5.295-10.17]	5.7[3.757-8.646]	<0.001
Province			
Kigali	1.3[0.902-1.817]	0.9[0.647-1.330]	0.684
South	1.6[1.230-2.113]	1.7[1.258-2.226]	<0.001
West	1.6[1.218-2.136]	1.6[1.208-2.238]	0.002
North	1.4[1.036-2.008]	1.5[1.051-2.086]	0.025
East	*Ref*	*Ref*	
Location			
Rural	*Ref*	*Ref*	
Urban	1.2[0.947-1.456]	1.3[1.012-1.612]	0.039
Education Level			
None	1.5[1.098-2.072]	1.1[0.812-1.599]	0.447
Primary School	1.1[0.775-1.454]	1.0[0.743-1.432]	0.849
Secondary and High School	*Ref*	*Ref*	
Marital Status			
Never married	*Ref*	*Ref*	
Currently married	2.2[1.621-3.018]	0.9[0.638-1.401]	0.782
Separated	3.6[2.580-5.096]	1.0[0.651-1.546]	0.986
Employment status			
Employed	*Ref*	*Ref*	
Unemployed	0.8[0.572-1.027]	1.2[0.857-1.604]	0.318
Ever consumed any Alcohol			
No	*Ref*	Ref	
Yes	1.5[1.131-1.897]	0.9[0.656-1.267]	0.584
Consumed alcohol with 12 months			
No	*Ref*	*Ref*	
Yes	1.4[1.135-1.686]	1.4[1.110-1.871]	0.006
Blood pressure measurement history			
No	*Ref*	*Ref*	
Yes	1.5[1.206-1.827]	1.1[0.840-1.312]	0.664
Cholesterol measurement history			
No	*Ref*	*Ref*	
Yes	2.6[1.450-4.709]	1.8[0.858-3.900]	0.117
Active transport (Walking/Bicycle use)			
Yes	*Ref*	*Ref*	
No	1.5[0.936-2.399]	1.1[0.870-2.233]	0.167
Perceived importance of salt reduction			
Very important	*Ref*	*Ref*	
Somewhat important	0.9[0.668-1.143]	0.9[0.711-1.253]	0.692
Not at all important	0.7[0.473-0.948]	0.7[0.517-1.077]	0.118
Total cholesterol levels			
Normal	*Ref*	*Ref*	
Borderline high	2.9[1.644-5.432]	1.8[0.958-3.241]	0.068
High	1.3[0.513-3.340]	1.3[0.430-3.741]	0.665
Physical active level			
Physically active	*Ref*	*Ref*	
Physical Inactive	1.2[0.767-1.733]	0.9[0.567-1.373]	0.580
Excessive drinking			
No excessive drinking	*Ref*	*Ref*	
excessive drinking	1.7[1.278-2.307]	1.4[0.982-1.940]	0.063
BMI Category			
Normal Weight	*Ref*	*Ref*	
Underweight	0.9[0.650-1.265]	0.7[0.522-1.060]	0.102
Overweight/Obese	1.7[1.418-2.142]	1.6[1.202-2.002]	0.001
Diabetes status			
Diabetic	4.9[3.476-6.929]	6.7[3.791-11.66]	<0.001
Non-diabetic	*Ref*	*Ref*	
Engagement in vigorous physical activity			
Yes	*Ref*	*Ref*	
No	1.7[1.283-2.193]	1.1[0.777-1.486]	0.660

### Multivariate analysis

Multivariate analysis identified factors associated with hypertension (Table 4). Age showed increasing risk: 30–44 years (AOR = 1.9, *p* < 0.001), 45–59 years (AOR = 4.1, *p* < 0.001), and 60–69 years (AOR = 5.7, *p* < 0.001) compared to 18–29 years. By province, Southern (AOR = 1.7, *p* < 0.001), Western (AOR = 1.6, *p* = 0.002), and Northern provinces (AOR = 1.5, *p* = 0.025) showed higher risk than Eastern province, while Kigali showed no difference (*p* = 0.684). Urban residents had higher odds than rural dwellers (AOR = 1.3, *p* = 0.039). Being overweight/obese increased odds (AOR = 1.6, *p* = 0.001) while underweight status showed no difference (*p* = 0.102). Diabetes had the strongest association (AOR = 6.7, *p* < 0.001), and Alcohol consumption in the past 12 months increased risk (AOR = 1.4, *p* = 0.006). Education level, marital status, employment status, and history of alcohol consumption, blood pressure measurement history, cholesterol measurement history, active transport, salt reduction importance, physical activity level, excessive drinking, and Vigorous physical activity showed no significant association, and were not significantly associated with hypertension, with *p* > 0.05.

## Discussion

This study examined key determinants of hypertension, highlighting significant associations with diabetes, age, BMI status, geographic location leaning toward urban areas, alcohol consumption, cholesterol levels, and blood glucose levels. These findings contribute to the literature on hypertension risk factors and emphasize the importance of targeted interventions. Addressing modifiable risk factors such as obesity, alcohol consumption, and cholesterol management can significantly reduce the incidence of hypertension and its associated complications.

Our findings from the 2022 STEPS Survey indicate notable shifts in hypertension prevalence and associated risk factors compared to the 2013 STEPS Survey. In 2013, the national prevalence of raised blood pressure was reported at 15.3%, increasing with age to about 40% among individuals aged 55–64 years. The prevalence of hypertension observed in this study is consistent with findings from national and global reports indicating an increasing burden of hypertension in low- and middle-income countries (LMICs)^14^. These findings suggest that hypertension remains a significant public health concern requiring urgent intervention. The high prevalence of hypertension in the study population reflects lifestyle and metabolic changes that may have contributed to the increasing incidence of NCDs. Previous studies have identified inadequate screening and diagnosis as challenges in hypertension management in Rwanda and other LMICs^15,16^. While screening is an important component of hypertension control, evidence on its effectiveness varies across different settings in sub-Saharan Africa. Recent studies suggest that screening alone may not translate into better outcomes unless it is embedded within broader systems of care. For instance, evidence from Malawi shows that improved diagnosis and treatment were primarily driven by access to structured referral pathways rather than behavior change following screening. Similarly, findings from South Africa emphasize that the design of screening, such as referral thresholds and timing, can substantially influence outcomes by minimizing false positives and improving follow-up^17,18^. In Rwanda’s context, strengthening hypertension screening should go hand in hand with comprehensive primary healthcare approaches that include accessible treatment options, long-term monitoring, and community-based support systems. To effectively reduce hypertension-related complications, screening programs must also address barriers to care continuity, medication adherence, and lifestyle modification, ensuring that detection leads to sustained management.

The 2022 survey shows a slight rise to 16.8%, with a similar age-related increase but a higher adjusted odds ratio for older adults, suggesting a persistent and growing burden among aging populations. Age was a strong predictor of hypertension, with older individuals exhibiting significantly greater odds of developing the condition. These findings align with global evidence that hypertension risk increases with age due to vascular remodeling, arterial stiffness, and cumulative exposure to risk factors such as poor diet and sedentary lifestyles^19,20^. Studies in sub-Saharan Africa (SSA) have shown that aging populations are experiencing a rapid increase in hypertension incidence due to epidemiological and lifestyle transitions^21,22^. Given these findings, age-specific interventions, including regular blood pressure monitoring and lifestyle modifications, should be emphasized to reduce the hypertension burden in aging populations. Additionally, overweight and obesity were identified in 17.1% of participants in 2013, whereas the 2022 findings report 20.6%, indicating a gradual increase in metabolic risk factors. These differences may reflect ongoing urbanization, dietary transitions, and sedentary lifestyles^9,23^.

The study revealed significant geographic disparities, with higher odds of hypertension in the western and southern provinces. This variation suggests regional differences in access to healthcare, lifestyle behaviors, and socioeconomic factors. Similar disparities have been reported in previous research, indicating that rural and semiurban populations often have limited access to hypertension diagnosis and treatment services^24^. Environmental and dietary factors may also contribute to these differences. Public health initiatives should prioritize improving healthcare access and promoting hypertension awareness in high-risk regions to address these disparities effectively.

Alcohol consumption in the past 12 months was significantly associated with hypertension. This finding aligns with existing research demonstrating that chronic alcohol intake can lead to increased blood pressure through mechanisms such as sympathetic nervous system activation and endothelial dysfunction^25–27^. While some studies suggest a protective effect of moderate alcohol consumption, excessive alcohol intake remains a well-established risk factor for hypertension^28,29^. Public health interventions should focus on alcohol reduction programs and awareness campaigns to mitigate their impact on cardiovascular health.

Borderline high cholesterol levels were associated with increased hypertension risk, supporting previous findings on the interplay between dyslipidemia and hypertension. Elevated cholesterol contributes to endothelial dysfunction and arterial stiffness, increasing vascular resistance and promoting hypertension development^30,31^. Studies have shown that lipid abnormalities are commonly found in hypertensive patients, reinforcing the need for cholesterol management as part of hypertension prevention strategies^32^. Strengthening cholesterol screening and lipid management programs in primary healthcare settings could help reduce hypertension-related complications.

Being overweight or obese significantly increased the odds of hypertension. This finding is consistent with global evidence linking obesity to hypertension through mechanisms such as insulin resistance, inflammation, and increased sympathetic nervous system activity^33,34^. The increasing prevalence of obesity in SSA due to urbanization and dietary changes has been identified as a key driver of hypertension and other metabolic disorders^35,36^. Public health interventions promoting healthy eating, physical activity, and weight management should be prioritized to curb the increasing burden of hypertension.

Diabetes was the strongest predictor of hypertension in this study, which is consistent with extensive research linking hypertension and diabetes through shared pathophysiological mechanisms, including insulin resistance and endothelial dysfunction^37,38^. The coexistence of hypertension and diabetes significantly increases the risk of cardiovascular events and mortality^39,40^. Integrated management approaches, such as routine screening for both conditions and lifestyle modification programs, should be implemented to reduce the impact of hypertension among diabetic individuals.

High blood glucose levels are inversely associated with hypertension, a finding that may reflect complex physiological interactions or the potential effect of medication use among diabetic patients. Previous studies have reported similar inverse associations, suggesting that some diabetic medications, particularly those that target insulin sensitivity, may lower blood pressure^39,41^. Further research is needed to clarify the relationship between glycemic control and long-term hypertension risk, considering the impact of glucose-lowering therapies on blood pressure regulation.

This study has several strengths, including its large nationally representative sample and comprehensive analysis of hypertension determinants. However, limitations should be acknowledged. The cross-sectional design captures associations at a single time point, making it difficult to determine whether risk factors precede hypertension development or result from it. Self-reported behavioral data, such as alcohol consumption, may be subject to recall bias. Additionally, our analysis did not fully explore several potential confounding factors, including detailed dietary patterns (particularly sodium intake), stress levels, and genetic predispositions, which may influence hypertension risk. Future studies employing mixed methods and repeated measurements over time would help clarify the directionality of observed associations and better characterize the complex interplay between modifiable risk factors and hypertension in Rwanda.

## Conclusion

This study highlights the burden and key determinants of hypertension in Rwanda. Age and obesity emerged as the strongest predictors, with older adults significantly more likely to have hypertension and overweight/obese individuals at higher risk compared to those with normal weight. While urban residents showed a slightly greater prevalence, the difference was not statistically significant after adjustment. Although factors such as alcohol consumption and high cholesterol levels were associated in the bivariate analysis, they were not significant in the multivariate model, suggesting potential confounding effects. These findings emphasize the urgent need for targeted public health interventions, including early screening, weight management, and lifestyle modifications, to address the growing burden of hypertension. Strengthening community-based prevention programs and implementing policy-driven strategies will be essential for improving cardiovascular health outcomes in Rwanda. Future research should focus on long-term trends and the effectiveness of intervention programs to support sustainable hypertension control measures.

## Supplementary Information

Below is the link to the electronic supplementary material.

Supplementary Material 1

## Data Availability

The data used in this analysis are from the 2022 Rwanda STEPs Survey. The authors do not own these data and are not the primary custodians. The data can be accessed upon request from the Rwanda Ministry of Health/Rwanda Biomedical Center, the University of Rwanda, or the WHO repository of STEPs Studies. Researchers interested in using these data should direct their requests to these institutions.

## Abbreviations

AOR: Adjusted odds ratio
BMI: Body mass index
CI: Confidence interval
COR: Crude odds ratio
DBP: Diastolic blood pressure
NCDs: Noncommunicable diseases
RNEC: Rwanda national ethics committee
SBP: Systolic blood pressure
SSA: Sub-Saharan Africa
WHO: World health organization
