# Supplementary material for: Prevalence and determinants of hypertension in rwanda: a secondary data analysis using the WHO STEPS survey 2022
Source: Sci Rep. 2025 Aug 23;15:31036. doi: 10.1038/s41598-025-14138-1 (PMC12375078; doi:10.1038/s41598-025-14138-1)
Supplement: Supplementary file 1 — Supplementary Material 1 [file 41598_2025_14138_MOESM1_ESM.docx]

| **Table 4:** Relationship between Sociodemographic, lifestyle and behavioral, biochemical measurements, health outcomes, and medical history factors and hypertension (n=5,676) | | | | |
| --- | --- | --- | --- | --- |
| **Variable** | Total weighted Frequency (n) | No Hypertension % [95% CI] | Hypertension % [95% CI] | P-value |
| Age group |  |  |  |  |
| 18-29 | 2,474,935 | 91.6[89.7,93.1] | 8.4[6.9,10.3] | <0.001 |
| 30-44 | 1,744,241 | 82.7[80.7,84.5] | 17.3[15.5,19.3] |  |
| 45-59 | 914,365 | 71.6[68.2,74.7] | 28.4[25.3,31.8] |  |
| 60-69 | 353,096 | 58.3[53.5,62.9] | 41.7[37.1,46.5] |  |
| Province |  |  |  |  |
| Kigali | 803,284 | 82.4[78.4,85.7] | 17.6[14.3,21.6] | 0.001 |
| South | 1,195,731 | 80.5[77.7,83.1] | 19.5[16.9,22.3] |  |
| West | 1,250,425 | 82.7[80.1,85.0] | 17.3[15.0,19.9] |  |
| North | 945,824 | 81.8[78.6,84.6] | 18.2[15.4,21.4] |  |
| East | 1,291,374 | 88[85.7,90.0] | 12[10.0,14.3] |  |
| Location |  |  |  |  |
| Urban | 1,002,729 | 80.5[77.5,83.2] | 19.5[16.8,22.5] | 0.026 |
| Rural | 4,483,908 | 83.9[82.5,85.2] | 16.1[14.8,17.5] |  |
| Sex |  |  |  |  |
| Male | 2,738,354 | 84.1[82.1,85.9] | 15.9[14.1,17.9] | 0.178 |
| Female | 2,748,282 | 82.5[80.9,83.9] | 17.5[16.1,19.1] |  |
| Education level |  |  |  |  |
| None | 1,611,896 | 79.9[77.6,82.0] | 20.1[18.0,22.4] | 0.003 |
| Primary School | 2,863,639 | 84.7[82.9,86.3] | 15.3[13.7,17.1] |  |
| Secondary and High School | 987,444 | 84.9[81.6,87.8] | 15.1[12.2,18.4] |  |
| Marital status |  |  |  |  |
| Never married | 1,725,003 | 90.6[88.3,92.5] | 9.4[7.5,11.7] | <0.001 |
| Currently married | 3,246,290 | 81.2[79.5,82.8] | 18.8[17.2,20.5] |  |
| Separated | 513,678 | 71.8[68.4,74.9] | 28.2[25.1,31.6] |  |
| Employment status |  |  |  |  |
| Employed | 4,387,180 | 82.4[81.0,83.8] | 17.6[16.2,19.0] | 0.011 |
| Unemployed | 1,099,457 | 86.6[83.8,89.0] | 13.4[11.0,16.2] |  |
| Current tobacco use |  |  |  |  |
| No | 5,099,829 | 83.4[82.0,84.6] | 16.6[15.4,18.0] | 0.530 |
| Yes | 386,808 | 82.1[78.0,85.6] | 17.9[14.4,22.0] |  |
| Ever consumed alcohol |  |  |  |  |
| No | 1,250,172 | 87.3[84.7,89.5] | 12.7[10.5,15.3] | 0.001 |
| Yes | 4,236,465 | 82.1[80.6,83.5] | 17.9[16.5,19.4] |  |
| Consumed alcohol within the past 12 months |  |  |  |  |
| No | 2,361,856 | 85.2[83.3,86.9] | 14.8[13.1,16.7] | 0.008 |
| Yes | 3,124,780 | 81.8[80.0,83.5] | 18.2[16.5,20.0] |  |
| Blood pressure measurement history |  |  |  |  |
| No | 2,855,786 | 86.2[84.5,87.7] | 13.8[12.3,15.5] | <0.001 |
| Yes | 2,630,851 | 80.1[78.2,81.9] | 19.9[18.1,21.8] |  |
| Cholesterol measurement history |  |  |  |  |
| No | 5,357,378 | 83.8[82.5,85.0] | 16.2[15.0,17.5] | <0.001 |
| Yes | 129,259 | 62.8[49.4,74.4] | 37.2[25.6,50.6] |  |
| Active Transport (walking/Bicycle use) |  |  |  |  |
| Yes | 5,300,037 | 83.4[82.0,84.6] | 16.6[15.4,18.0] | 0.402 |
| No | 186,600 | 80.6[73.1,86.4] | 19.4[13.6,26.9] |  |
| Perceived importance of salt reduction |  |  |  |  |
| Very important | 1,096,509 | 82.2[79.1,84.9] | 17.8[15.1,20.9] | 0.253 |
| Somewhat important | 3,392,467 | 83[81.4,84.5] | 17[15.5,18.6] |  |
| Not at all important | 997,661 | 85.3[82.4,87.8] | 14.7[12.2,17.6] |  |
| Total cholesterol levels |  |  |  |  |
| Normal | 5,375,228 | 83.4[82.2,84.7] | 16.6[15.3,17.8] | 0.034 |
| Borderline high | 59,685 | 68.9[56.8,78.8] | 31.1[21.2,43.2] |  |
| High | 51,724 | 82[64.3,92.1] | 18[7.9,35.7] |  |
| Fruits consumption frequency |  |  |  |  |
| None | 1,658,734 | 82.4[80.2,84.5] | 17.6[15.5,19.8] | 0.521 |
| 1-3 days | 3,033,936 | 84[82.3,85.5] | 16[14.5,17.7] |  |
| 4-7 days | 752,137 | 82.5[78.6,85.9] | 17.5[14.1,21.4] |  |
| Don't know | 41,830 | 78.4[62.7,88.7] | 21.6[11.3,37.3] |  |
| Physical activity level |  |  |  |  |
| Physical Inactive | 250,582 | 79.5[72.9,84.8] | 20.5[15.2,27.1] | 0.169 |
| Physically active | 5,236,055 | 83.5[82.1,84.7] | 16.5[15.3,17.9] |  |
| Excessive drinking |  |  |  |  |
| No | 5,096,398 | 83.7[82.4,84.9] | 16.3[15.1,17.6] | 0.004 |
| Yes | 390,239 | 77.5[72.4,81.8] | 22.5[18.2,27.6] |  |
| BMI category |  |  |  |  |
| Underweight | 569,535 | 86.9[82.9,90.1] | 13.1[9.9,17.1] | <0.001 |
| Normal Weight | 3,910,577 | 85.2[83.7,86.6] | 14.8[13.4,16.3] |  |
| Overweight/Obese | 1,006,525 | 73.6[70.4,76.6] | 26.4[23.4,29.6] |  |
| Vegetable consumption |  |  |  |  |
| Inadequate | 2,299,330 | 83[81.1,84.8] | 17[15.2,18.9] | 0.306 |
| Adequate | 1,441,160 | 84.6[82.1,86.8] | 15.4[13.2,17.9] |  |
| Diabetic status |  |  |  |  |
| Non-diabetic | 5,276,905 | 84.4[83.1,85.6] | 15.6[14.4,16.9] | <0.001 |
| Diabetic | 209,732 | 56[48.0,63.8] | 44[36.2,52.0] |  |
| High | 129,684 | 70.1[60.3,78.3] | 29.9[21.7,39.7] |  |
| History of Stroke |  |  |  |  |
| Yes | 65,150 | 76.1[51.9,90.3] | 23.9[9.7,48.1] | 0.405 |
| No | 5,421,487 | 83.4[82.1,84.6] | 16.6[15.4,17.9] |  |
| History of heart attack or angina |  |  |  |  |
| Yes | 220,146 | 78.4[72.1,83.6] | 21.6[16.4,27.9] | 0.065 |
| No | 5,266,491 | 83.5[82.1,84.7] | 16.5[15.3,17.9] |  |
| Recent cholesterol treatment |  |  |  |  |
| No | 5,475,463 | 83.3[82.0,84.5] | 16.7[15.5,18.0] | 0.601 |
| Yes | 11,174 | 76[36.6,94.6] | 24[5.4,63.4] |  |
| Engagement in vigorous physical activity |  |  |  |  |
| Yes | 1,299,094 | 89.4[86.9,91.5] | 10.6[8.5,13.1] | <0.001 |
| No | 4,187,543 | 81.4[79.8,82.8] | 18.6[17.2,20.2] |  |
